# A Multi-Site Study of Traumatic Brain Injury in Mexico and Colombia: Longitudinal Mediational and Cross-Lagged Models of Family Dynamics, Coping, and Health-Related Quality of Life

**DOI:** 10.3390/ijerph17176045

**Published:** 2020-08-20

**Authors:** Annahir N. Cariello, Paul B. Perrin, Yaneth Rodríguez-Agudelo, Silvia Leonor Olivera Plaza, Maria Cristina Quijano-Martinez, Juan Carlos Arango-Lasprilla

**Affiliations:** 1 Virginia Commonwealth University, 907 Floyd Ave, Richmond, VA 23284, USA; carielloa@mymail.vcu.edu (A.N.C.); pperrin@vcu.edu (P.B.P.); 2 Neuropsychology Department, National Institute of Neurology and Neurosurgery, Mexico City 14269, Mexico; yaneth_r@hotmail.com; 3 Grupo de Investigación Carlos Finlay, Universidad Surcolombiana, Neiva, Huila, Colombia; silvileo06@hotmail.com; 4 Department of Social Sciences, Pontificia University of Javeriana-Cali, Cali, Colombia; mcquijano@javerianacali.edu.co; 5 BioCruces Bizkaia Health Research Institute, Cruces University Hospital Barakaldo, 48903 Barakaldo, Spain; 6 IKERBASQUE, Basque Foundation for Science, 48013 Bilbao, Spain; 7 Department of Cell Biology and Histology, University of the Basque Country (UPV/EHU), 48940 Leioa, Biscay, Spain

**Keywords:** traumatic brain injury, health-related quality of life, family dynamics, coping

## Abstract

Latin America has high rates of traumatic brain injury (TBI), yet reduced mental and physical health outcomes due to limited rehabilitation services. To understand the psychosocial adjustment process in TBI patients in Latin America, the incorporation of cultural values including family-based variables is imperative. The current study examined relations among healthy family dynamics, coping, and mental and physical health related quality of life (HRQL) among a sample of TBI patients across three sites and two countries over the first 4 months post-injury. A sample of 109 acute TBI patients from Mexico and Colombia were recruited, and a series of longitudinal mediation and cross-lag panel analyses were run. Mental and physical HRQL were positively associated with each other, as well as healthy family dynamics and coping. Coping partially mediated the effects of healthy family dynamics on both mental and physical HRQL. Family dynamics showed the strongest cross-lag relationships with coping going from 2 months to 4 months. Similarly, coping showed the strongest cross-lag relationships with both mental and physical HRQL going from baseline to 2 months. Results provide empirical support for the importance of a rehabilitation workforce that has been trained in and can effectively implement family-based interventions for individuals with TBI in Latin America.

## 1. Introduction

Traumatic brain injury (TBI) is the greatest global contributor to death and disability among all trauma-related injuries, and has been referred to as the “silent epidemic”, since its lasting impairments are often invisible [1]. As Latin America has the highest incidence of TBI due to violence and road traffic injuries, TBI is a pressing public health and medical problem in the region [2,3]. In Mexico, TBI is the third leading cause of death [4]. Colombia has higher than average rates of intentional injuries, due to hostile guerilla warfare and landmine explosions resulting in high rates of TBI [5]. In Latin America, Ramachandran and associates [6] note the lack of standardized protocols for trauma assessment and management as a major limitation in medical resources affecting TBI morbidity and mortality rates, as the establishment of trauma protocols standardizing care across medical institutions in North America, Europe, and Australia led to a 15–20% reduction in the risk of death from TBI [7]. Compared to other regions, the effects of TBI are especially severe in Latin America, due to these and other limited medical and rehabilitation resources [8,9].

The presentation, severity, and duration of physical health complications vary from patient to patient, due to injury-related variables and injury severity [10]. As TBI creates multiple permanent and transient impairments, health related quality of life (HRQL)—which comprises one’s sense of well-being, as well as life satisfaction, including psychological, physical and social functioning, and perceptions of independence, self-efficacy, self-concept, and social support [11]—is a critical outcome reflecting patient perceived recovery [12]. TBI patients reported lower levels of HRQL compared to the general population [13], and role limitations-physical and emotional domains of HRQL have been shown to be some of the lowest reported domains after TBI [14]. Cognitive impairments among TBI patients may impact social relationships, and thereby result in increased emotional distress [15]. Investigating HRQL 10 years post-injury, Horneman and associates [16] found functional limitations and emotional problems to be the most salient impairments for patients. Significant factors predicting HRQL have included pain and disability status after TBI [17]. Understanding HRQL in TBI patients and factors that impact prognosis can serve as a guide for caregivers, family members, and the rehabilitation workforce.

The primary responsibility for lifelong well-being of TBI patients often falls on immediate family, and therefore TBI not only affects the patient, but the entire familial system [18]. Family members aid TBI patients in performing everyday tasks, requiring a large amount of time and resources [9]. Due to the reductions in TBI patients’ functioning, typically, various family members including spouses, parents, children, and siblings play a role in intensive and lifelong care [19]. TBI patients’ emotional, physical, and behavior changes necessitate family members to adapt and cope, yet, as most TBIs are sudden, most families are unprepared [20].

In Latin America specifically, family dynamics have been shown to impact the TBI rehabilitation process and adjustment, likely in part because of cultural values including familialism [21]. Familialism, or familismo, refers to Latinos’ identity as part of a family, and Latinos’ families and communities often provide significant support [22]. Norup and colleagues found that TBI caregivers in Mexico reported higher unmet needs for emotional support and instrumental support than caregivers in several other global regions, suggesting a high importance of and desire for family connection and support [23]. Similarly, Coy and associates found that the effect of TBI social impairments on caregiver burden in Mexico was lessened if caregivers reported strong family functioning [24].

As familism is a core value in Latin American families and impacts TBI rehabilitation, the TBI rehabilitation workforce has only recently begun to use a family-systems approach to understand family dynamics in the context of TBI [25]. Family-system theory emphasizes the ways in which the impact of a TBI can reverberate through all members of the family, drawing attention to the inherent strain and sudden changes TBI can place on family members [26]. As TBI is considered to be a family condition, further investigation of how family dynamics impact TBI patients’ mental and physical HRQL in Latin American countries is needed, and particularly though a longitudinal approach.

Sense of coherence (SOC) may be critical in TBI patient adjustment, and refers to internal resources that enable people to cope with stressful situations, thereby maintaining psychological and physical health. SOC embodies three mutually interacting components—comprehensibility, manageability and meaningfulness—that contribute to an individual’s perception of the world and facilitate adaptive coping [27]. Although the association between SOC and adjustment to disabilities has been extremely well supported [28,29], scarce research has been conducted on SOC in TBI. Investigating SOC 6–15 years after TBI, Jacobsson, Westerberg, Malec, and Lexell found SOC was strongly associated with life satisfaction [30]. Similarly, Collicutt McGrath and Linley found TBI patients maintained a stable SOC in the face of the many threats posed by brain injury [31]. SOC may possibly explain individual differences in coping with stressful disability situations, and thereby may be of vital importance in investigating the challenges immediately after sustaining a TBI [27,32].

Latin America has elevated rates of TBI, yet little research has been conducted on predictors of patient HRQL in the region. The existing research has generally focused on problems and unmet needs of families affected by TBI, examining poor family functioning and insufficient coping resources. Furthermore, very little research has examined the particular cultural strengths that may improve TBI patient mental and physical health outcomes in Latin America, and no studies have been conducted on the relationship between healthy family dynamics and TBI patient HRQL, despite the vast empirical support documenting the importance of familialism in this population. As a result, the purpose of the current study was to examine the relations among healthy family dynamics, patient SOC, and patient mental and physical HRQL among a sample of TBI patients across three sites and two countries in Latin America over the first four months after injury. It was hypothesized that: (a) healthy family dynamics at baseline (before hospital discharge) would have a direct and positive effect on patient SOC (at 2 months); (b) patient SOC (at 2 months) would have a direct and positive effect on patient mental and physical HRQL (at 4 months); (c) healthy family dynamics at baseline (before hospital discharge) would have a direct and positive effect on patient mental and physical HRQL (at 4 months); and (d) healthy family dynamics at baseline (before hospital discharge) would have an indirect effect on patient mental and physical HRQL (at 4 months) through patient SOC (at 2 months). It was also hypothesized that in a cross-lagged panel design: (a) in the longitudinal relationship between family dynamics and SOC, family dynamics would consistently show larger cross-lag relationships (i.e., causal preponderance [33]) between the variables going from baseline to 2 months and 2 months to 4 months; and (b) in the longitudinal relationship between SOC and mental and physical HRQL, SOC would consistently show larger cross-lag relationships (i.e., causal preponderance [33]) between the variables going from baseline to 2 months and 2 months to 4 months.

## 2. Materials and Methods

### 2.1. Participants

A sample of 109 individuals with TBI were recruited from three hospitals in Mexico City, Mexico, and in Cali and Neiva, Colombia. These hospitals have high volumes of TBI treatment that aided in recruitment for the project. There were a number of inclusion criteria for participants: (a) participants must have been hospitalized at the time of recruitment with a diagnosed acute TBI; (b) participants must have been be over the age of 18; and (c) participants must have been able speak in Spanish. Individuals with TBI were excluded if they: (a) did not have or need a caregiver; (b) were not discharged directly home (i.e., discharged to a nursing home or living facility); or (c) had a history of other neurological conditions, serious psychiatric disorders, alcohol and drug abuse, or developmental or learning disabilities. To ensure participants met these criteria, they were pre-screened prior to beginning the study. Participants’ demographics appear in Table 1.

### 2.2. Measures

Individuals with TBI completed the following measures orally with a research assistant at three time points: before hospital discharge, 2 months after discharge, and 4 months after discharge.

#### 2.2.1. Short Form Health Survey (SF-36)

The Spanish SF-36 is one of the most widely used instruments to assess self-reported health-related quality of life (HRQL) in individuals with TBI [34]. The items assess eight health concepts that can be combined into a raw physical health composite score and mental health composite score, both of which were used in the current study. Scores range from 0–100, with higher scores reflect greater HRQL. The SF-36 has well-established validity by Cronbach’s alpha ranging between 0.70 and 0.90 in Spanish-speaking populations [35]. In the current sample, the Cronbach’s alpha for Physical HRQL composite score for Time 1 was 0.93, Time 2 was 0.90, and Time 3 was 0.93. The Cronbach’s alpha for the mental HRQL composite score for Time 1 was 0.90, Time 2 was 0.94, and Time 3 was 0.90.

#### 2.2.2. Sense of Coherence Scale (SOC-13)

The Spanish SOC-13 is a 13-item self-report questionnaire that assesses a person’s tendency to perceive life events as meaningful, understandable, and manageable [36,37]. Scoring uses the mean of all items, and higher scores reflect a stronger sense of coherence [30]. The SOC has good internal validity by Cronbach’s alpha of 0.80 in Spanish-speaking populations [37] and has been validated in TBI patients with a strong Cronbach’s alpha of 0.88 [30]. In the current sample, the Cronbach’s alpha for SOC for Time 1 was 0.76, Time 2 was 0.78, and Time 3 was 0.80.

#### 2.2.3. Family Assessment Device (FAD)

The Spanish FAD is a widely used self-report measure designed to assess family functioning. The FAD measures the overall level of family functioning. Typically, higher scores indicate more pathology in the family system, but for the purposes of this study wherein healthy family dynamics were emphasized, an inverse of the overall scores was used and therefore higher scores indicated healthier dynamics. The Spanish FAD has good internal reliability in with a Cronbach’s alpha = 0.82. [38]. In the current sample, the Cronbach’s alpha for FAD for Time 1 was 0.88, Time 2 was 0.85, and Time 3 was 0.82.

### 2.3. Procedure

This study was a part of a larger parent study that was approved by the Virginia Commonwealth University Institutional Review Board (approval code HM20001660), as well as at each of the sites in Latin America. Participants who met criteria were asked for informed consent to participate under ethics committee approval at each site. Upon completion of the consent form, participants completed the questionnaires and demographic information orally, and responded to items read aloud by a research assistant. After completion of the survey at each time point (before hospital discharge, 2 months after discharge, and 4 months after discharge), participants were paid an incentive of USD 10 cash (or the equivalent in their country of origin).

### 2.4. Data Analysis Plan

#### 2.4.1. Preliminary Analyses

To examine bivariate correlations among family dynamics, SOC, HRQL at each of the time points, a Pearson product moment correlation matrix was created.

#### 2.4.2. Primary Analyses

Two meditational models were developed and tested using Amos (SPSS, IBM, Armonk, NY, USA; Version 26.0). This statistical approach examined direct and indirect effects using 2000 bootstrap samples. Each meditational model was specified for each HRQL composite score (i.e., physical and mental). Healthy family dynamics was measured using the FAD, sense of coherence by the SOC-13, and the mental and physical HRQL composite scores were comprised of the 4 subscales of the SF-36 within these constructs. Direct and indirect relationships between healthy family dynamics at baseline, SOC at 2 months, and HRQL at 4 months were examined. The same statistical procedure was performed for mental HRQL as the outcome variable. Three cross-lagged panel structural equation models (SEMs) were run with Amos (SPSS, IBM, Armonk, NY, USA; Version 26.0). A cross-lagged panel design is used to test causal preponderance of two variables in the relation between each other over time. In a cross-lagged panel, the same variable, or sets of variables, must be assessed at more than one time point. Path coefficients of the cross-paths between the variables or sets of variables over time were calculated, and the coefficient of cross-path that is larger in magnitude shows which variable is more causally preponderant in the relation between the two variables. In the current study, individuals with TBI completed measures of family dynamics and SOC at baseline, and 2 months and 4 months after injury. The same statistical procedure was performed for SOC and physical HRQL and for SOC and mental HRQL.

## 3. Results

For the measures in the study, the following percent of items were missing across all participants at each time point, respectively: HRQL Time 1(0.0%), HRQL Time 2 (1.0%), HRQL Time 3 (3.7%), SOC Time 1 (0.0%), SOC Time 2 (1.0%), SOC Time 3 (3.7%), FAD Time 1 (2.4%), FAD Time 2 (3.4%), and FAD Time 3 (7.0%). Missing data were imputed using the expectation maximization algorithm within each time point for items within a scale or across time points for a single scale if there were not enough items with complete data (<50%) for that scale at a single time point. In the correlation matrix (Table 2), most of the variables were associated with each other as would be expected, except for physical health related quality of life at Time 1.

### 3.1. Mediations

**Figure 1:**
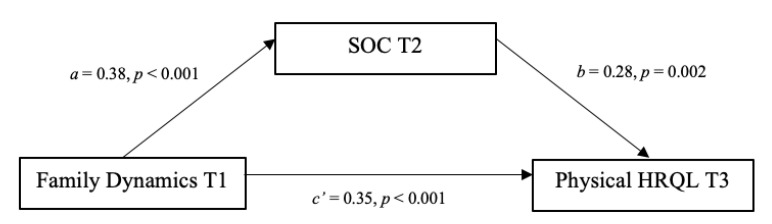
Statistical representation of sense of coherence (SOC) as a mediator of the relationship between family dynamics and patient physical health-related quality of life (HRQL). Note: SOC = Sense of Coherence; HRQL = Health-Related Quality of Life.

Two meditational models were run using Amos (Version 26.0) to examine patterns of relationships among physical HRQL, mental HRQL, SOC and healthy family dynamics. In the first mediational model (Figure 1, with standardized path coefficients), healthy family dynamics was specified to have a direct effect on physical HRQL, as well as an indirect effect through SOC, using 5000 bootstrap samples. The direct path from healthy family dynamics to SOC (β = 0.38 *p* < 0.001) was statistically significant. The direct path from healthy family dynamics to physical HRQL (β = 0.35, *p* < 0.001) was statistically significant, as well as the direct path from SOC to physical HRQL (β = 0.28, *p* = 0.002). Further, the indirect effect of healthy family dynamics on physical HRQL through SOC was statistically significant (β = 0.11, *p* = 0.004), indicating a partial mediation because the direct path from healthy family dynamics to physical HRQL (c’ path) was still statistically significant.

**Figure 2:**
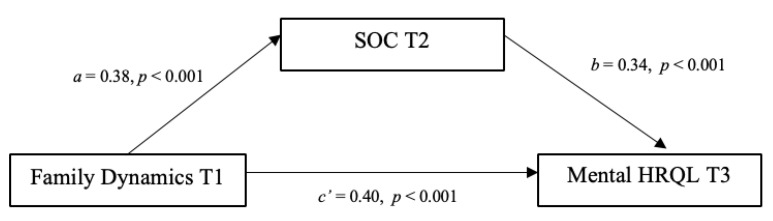
Statistical representation of SOC as a mediator of the relationship between family dynamics and patient mental HRQL. Note: SOC = Sense of Coherence; HRQL = Health-Related Quality of Life.

In the second mediational model (Figure 2, with standardized path coefficients), healthy family dynamics was specified to have a direct effect on mental HRQL, as well as an indirect effect through SOC, using 5000 bootstrap samples. The direct path from healthy family dynamics to SOC (β = 0.38, *p* < 0.001) was statistically significant. The direct path from healthy family dynamics to mental HRQL (β = 0.40, *p* < 0.001) was statistically significant as well as the direct path from SOC to mental HRQL (β = 0.34, *p* < 0.001). Further, the indirect effect of healthy family dynamics on mental HRQL through SOC was statistically significant (β = 0.13, *p* = 0.006), indicating a partial mediation because the direct path from healthy family dynamics to mental HRQL (c’ path) was still statistically significant.

### 3.2. Cross-Lagged Effects

Three cross-lagged panels were run using Amos (Version 26.0) to examine causal preponderance [33] in the relationships among physical HRQL, mental HRQL, SOC, and healthy family dynamics. The two variables in each model at Time 1 were allowed to correlate with each other. Disturbance (error) terms were calculated for all variables in the models at Times 2 and 3 which were allowed to correlate with each other within a time point, because endogenous variables cannot be specified to correlate.

**Figure 3:**
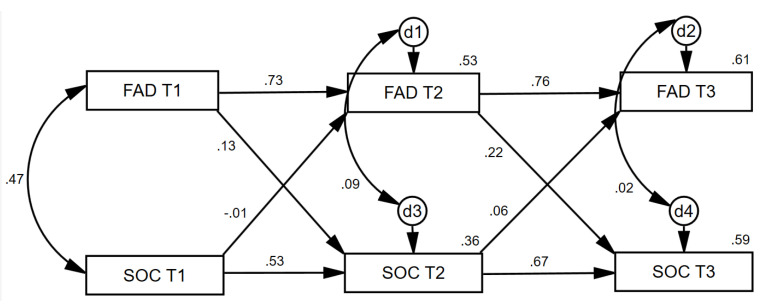
Statistical representation of longitudinal cross-lag relationships between family dynamics and patient SOC at baseline, 2 months, and 4 months. Note: FAD = Healthy Family Functioning; SOC = Sense of Coherence. d = Disturbance Term.

In the first cross-lagged panel analysis (Figure 3), the family dynamics variable at each time point was positively related to itself over time, as was SOC, as would be expected. Healthy family dynamics at Time 1 did not exert a unique effect (when controlling for SOC at Time 1) on SOC at Time 2 (β = 0.13, *p* = 0.135). Similarly, SOC at Time 1 did not exert a unique effect (when controlling for family dynamics at Time 1) on family dynamics at Time 2 (β = −0.01, *p* = 0.904). Healthy family dynamics at Time 2 did exert a unique effect (when controlling for SOC at Time 2) on SOC at Time 3 (β = 0.22, *p* < 0.001). Conversely, SOC at Time 2 did not exert a unique effect (when controlling for family dynamics at Time 2) on family dynamics at Time 3 (β = 0.06, *p* = 0.366). This pattern of relationships suggests that at least between Times 2 and 3, family dynamics exhibited more causal preponderance [33] in its relationship with SOC.

**Figure 4:**
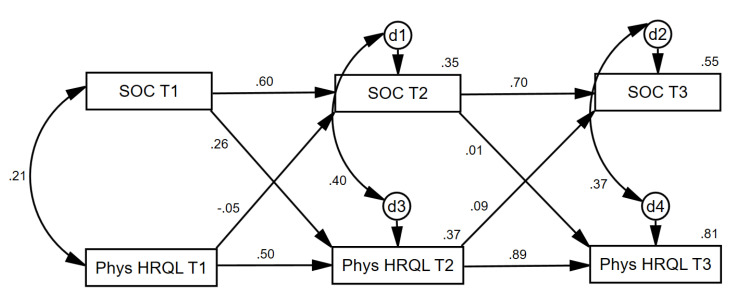
Statistical representation of longitudinal cross-lag relationships between patient SOC and patient physical HRQL at baseline, 2 months, and 4 months. Note: SOC = Sense of Coherence; Phys HRQL = Physical Health-Related Quality of Life. d = Disturbance Term.

In the second cross-lagged panel analysis (Figure 4), the SOC variable at each time point was positively related to itself over time, as was physical HRQL, as would be expected. SOC at Time 1 exerted a unique effect (when controlling for physical HRQL at Time 1) on physical HRQL at Time 2 (β = 0.26, *p* < 0.001). Conversely, physical HRQL at Time 1 did not exert a unique effect (when controlling for SOC at Time 1) on SOC at Time 2 (β = −0.05, *p* = 0.542). SOC at Time 2 did not exert a unique effect (when controlling for physical HRQL at Time 2) on physical HRQL at Time 3 (β = 0.01, *p* = 0.882). Similarly, physical HRQL at Time 2 did not exert a unique effect (when controlling for SOC at Time 2) on SOC at Time 3 (β = 0.09, *p* = 0.190). This pattern of relationships suggests that at least between Times 1 and 2, SOC exhibited more causal preponderance [33] in its relationship with physical HRQL.

**Figure 5:**
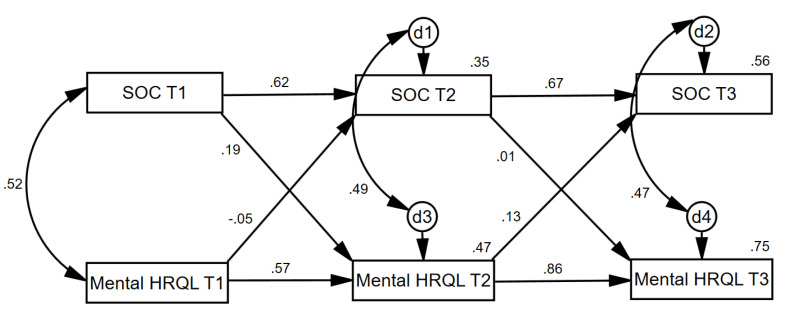
Statistical representation of longitudinal cross-lag relationships between patient SOC and patient mental HRQL at baseline, 2 months, and 4 months. Note: SOC = Sense of Coherence; Mental HRQL = Mental Health-Related Quality of Life. d = Disturbance Term.

In the third cross-lagged panel analysis (Figure 5), the SOC variable at each time point was positively related to itself over time, as was mental HRQL, as would be expected. SOC at Time 1 exerted a unique effect (when controlling for mental HRQL at Time 1) on mental HRQL at Time 2 (β = 0.19, *p* = 0.018). Conversely, mental HRQL at Time 1 did not exert a unique effect (when controlling for SOC at Time 1) on SOC at Time 2 (β = −0.05, *p* = 0.574). SOC at Time 2 did not exert a unique effect (when controlling for mental HRQL at Time 2) on mental HRQL at Time 3 (β = 0.01, *p* = 0.854). Similarly, mental HRQL at Time 2 did not exert a unique effect (when controlling for SOC at Time 2) on SOC at Time 3 (β = 0.13, *p* = 0.083). This pattern of relationships suggests that at least between Times 1 and 2, SOC exhibited more causal preponderance [33] in its relationship with mental HRQL.

## 4. Discussion

The purpose of this study was to examine the relationships among healthy family dynamics, SOC, and mental and physical HRQL among a sample of TBI patients across three sites and two countries in Latin America over the first four months after injury. A sample of 109 individuals with TBI were recruited from three hospitals in Mexico City, Mexico, and in Cali and Neiva, Colombia which produced a varied sample of individuals with TBI from diverse marriage, sex, age, and employment statuses. Congruent with the hypotheses, patient SOC partially mediated the effects of healthy family dynamics on both patient mental and physical HRQL over time. Family dynamics showed larger cross-lagged relationships with patient SOC going from 2 months to 4 months. Patient SOC showed larger cross-lagged relationships with both patient mental and physical HRQL going from baseline to 2 months. Results from this study provide empirical support for training rehabilitation professionals in Latin America in the integration of a family-systems approach to TBI rehabilitation.

### 4.1. Mediations

Patterns of relationships among healthy family dynamics, patient SOC, and patient mental and physical HRQL were examined using two mediational models. The first mediational model investigated the relationships among healthy family dynamics, patient SOC, and patient physical HRQL. Similar to previous research, the current study found a positive direct relationship between healthy family dynamics and patient SOC [39]. The current sample also found direct relationships between healthy family dynamics and patient physical HRQL, as well as from patient SOC to patient physical HRQL, which are also congruent and add to the previous literature. Andren and Elmståhl [40] found SOC was positively associated with better perceived health, and Gison and colleagues found SOC predicted HRQL [41]. Previous research also supports the link between increased family collaboration in the rehabilitation process and better TBI outcomes [42]. The current study adds to the literature by finding these associations in TBI patients and directly linking healthy family functioning and SOC to TBI mental and physical health outcomes.

Similarly, and as hypothesized, a partial indirect (mediational) relationship was found from healthy family dynamics to patient physical HRQL through patient SOC. Many factors may have contributed to these current findings, including the core Latin value of familism, congruent with Kouneski’s study, wherein families facing chronic illnesses that cope with family flexibility and cohesion promote positive coping behaviors [43]. The current study adds to the literature by linking healthy family dynamics to patient physical HRQL through patient SOC, reinforcing TBI rehabilitation as an injury that not only affects the patient but the entire family system, which, in turn, impacts TBI patient mental and physical health outcomes [18]. As patient SOC is only partially explained the relationship between healthy family dynamics and patient physical HRQL, other factors may be impacting patient physical HRQL post-injury including access to rehabilitation services including increased patient neurobehavioral problems and poorer functional outcomes found in Latinos 1 year post injury [8], which may possibly be due to limited medical and rehabilitation resources. This lack of resources may be impacting family dynamics and patient physical HRQL outcomes.

The second mediational model investigated the relationships among healthy family dynamics, patient SOC, and patient mental HRQL. Patient SOC partially mediated the relationship between healthy family dynamics and mental HRQL. A direct positive relationship between healthy family dynamics and patient mental HRQL is similar to previous findings and also adds substantially to the literature. Previous research has focused on the impact of TBI severity on caregivers’ mental health [44] or documented the presence of trauma-related psychiatric disorders in TBI patients [45]. No research to date has identified the connection between healthy family dynamics and positive mental HRQL outcomes in TBI patients. The direct relationship between patient SOC and patient mental HRQL is similar to previous findings including from Jacobsson, Westerberg, Malec, and Lexell [30], who found a strong association between SOC and life satisfaction 6–15 years post-injury, as well as Lammel’s study, wherein SOC had a direct and positive effect on psychosocial functioning [46].

The mediational effect of patient SOC in this model may in part explain the impact of healthy family dynamics on mental HRQL in TBI patients. These findings are congruent with Antonovsky’s theory, which postulated that a strong SOC affords an individual a powerful way of dealing with complex and difficult life situations, and influences emotional responses to stressful situations [27]. Similarly, the current findings add to the literature that supports SOC being associated with greater familial affection and easier communication [29]; thereby, SOC is partially accounting for the relationship between healthy family dynamics and TBI patient mental HRQL outcomes. As patient SOC only partially mediated the relationship, there may be other variables impacting this association. Another important Latino belief that may be impacting TBI mental HRQL outcomes is spirituality or religiosity. Lopez found spirituality may impact rehabilitation in Latinos such that an endurance of disability can be seen as trials or tribulation that demonstrate worthiness for a spiritual reward [47]. These cultural beliefs and values may impact the relationship between healthy family dynamics to mental HRQL through SOC, as TBI patients’ meaning making may be tied to their religiosity and spirituality, which has been found to be a buffer against depression and stress in Latinos [48]. Religiosity and social interactions outside of the family maybe affecting family functioning, TBI patient coping (SOC), and mental and physical HRQL.

### 4.2. Cross-Lagged Effects

The longitudinal relationships among family dynamics, patient SOC, and patient physical and mental HRQL were examined using three cross-lagged panels. In the first cross-lagged panel analysis, healthy family dynamics at Time 2 exerted a unique effect on SOC at Time 3. Conversely, patient SOC was not found to exert a unique effect on healthy family dynamics across time. This pattern of relationships suggests that between Times 2 and 3, family dynamics exhibits more causal preponderance [33] in its relationship with patient SOC. These results provide additional empirical support for the importance of familism as a driver of coping in TBI recovery. Healthy family functioning post-injury may facilitate the maintenance of patient SOC throughout recovery. This is again congruent with Latino familism, wherein there is a share sense of responsibility towards family members, as well as the previous literature, such that participation of the entire family helps foster a sense of mutual ownership of the TBI [49] may in turn be supporting TBI coping post-injury. In Mexico, Lehan and colleagues [49] discovered that both TBI patients and their family members who reported relatively high levels of family communication and satisfaction also reported greater levels of adaptability and cohesion (central to SOC).

In the second and third cross-lagged panel analysis, SOC at Time 1 exerted a unique effect on both patient physical and mental HRQL at Time 2. Conversely, both patient physical and mental HRQL were not found to exert any unique effects on patient SOC across time. This pattern of relationships suggests that between Times 1 and 2, patient SOC exhibits more causal preponderance [33] in its relationships with both patient mental and physical HRQL. These findings are congruent with Collicutt McGrath and Linley [31], who found patients maintained a stable SOC in the face of the many threats posed by brain injury. As SOC at Time 1 was found to affect both patient physical and mental HRQL at Time 2, the current study provides empirical support for the integration of cognitive behavioral mental health services during hospitalization. This is congruent with Snell and colleagues [50] who found coherent understanding of TBI and recovery resulted in decreased anxiety and facilitated self-esteem and self-control in TBI patients, resulting in positive recovery expectations. The current findings provided additional empirical support to Antonovsky’s assertions about SOC, such that increased comprehensibility, manageability, and meaningfulness resulted in better health outcomes in TBI patients [27]. Overall, these patterns of relationships suggest the importance of rehabilitation services throughout recovery that provide TBI patients with resources increasing their comprehensibility and manageability post-injury, and provide meaning to recovery.

### 4.3. Implications

The results of this study can help to inform TBI clinical interventions, research, and rehabilitation practices in Latin America and in attempts to improve TBI patient mental and physical health outcomes. As healthy family functioning at baseline was found to impact patient mental and physical HRQL of TBI patients at 4 months, especially through patient SOC at 2 months, the rehabilitation workforce in Latin America are encouraged to be trained in assessing the level of family functioning at baseline, as doing so may provide some insight to TBI patients’ adjustment over time. As patient SOC was found to be a significant mediator, rehabilitation services may benefit from targeted clinical interventions focused on the TBI patients’ comprehensibility, manageability, and meaningfulness, which may be contributing their coping post-injury. As family functioning over time was found to impact TBI patients’ SOC and HRQL more than the reverse causal direction, these clinical implications for targeting the family system gain even stronger support.

Due to these findings, implementation of clinical interventions to aid in the development and maintenance of healthy family functioning and patient SOC during TBI recovery and rehabilitation may improve patient mental and physical HRQL. Cognitive behavioral therapy (CBT) has been found to be efficacious for treatments among TBI patients with depression, anxiety, anger, and adjustment concerns [51]. Ashman and colleagues found CBT was efficacious in improving depression in TBI patients in the United States by engaging in cognitive restructuring, the creation of less self-critical dialogues, and focusing on the “here and now” [52]. CBT is highly structured and, as such, is well suited to address the combination of possible TBI presentations [53]. SOC is rooted in three general concepts of comprehensibility, manageability, and meaningfulness [27], which maps onto common CBT interventions well. The current study provides empirical support for the integration of CBT to aid in the development and maintenance of SOC throughout TBI recovery.

As family functioning was found to impact patient mental and physical HRQL, clinical assessment of family functioning at injury as well as throughout rehabilitation may aid TBI recovery post-injury. Stejskal [54] found marriage and family therapy (MFT) and family interventions were efficacious in fostering a mutual ownership of the TBI, wherein the TBI patient is not considered to be the problem, and the therapist observes family patterns firsthand and intervenes in the moment. Family therapy sessions are focused on both the family and TBI patient with the goals of healing as well as providing support for adjustment to the TBI [55]. An integrated care approach is recommended, wherein the entire rehabilitation team supports the family, as family members may have specific and disparate individual needs [56].

### 4.4. Limitations and Future Directions

The current findings are recommended to be considered within the context of several limitations. First, data were collected at hospitalization, 2 months, and 4 months post-injury and as such provide limited insight on the long-term effects of TBI on family systems. As TBI requires consistent support and caregiving by family members to manage medical treatments and rehabilitation, as well as aiding TBI patients in preforming everyday tasks [9], long-term effects of this dynamic on the family system is necessary to provide additional support to the current findings. Future studies are encouraged to investigate these variables at 6 months, 1 year, 2 years, 5 years, and 10 years, to aid in the understanding of family systems on TBI patient HRQL. Additionally, the sample was recruited from Mexico and Colombia, and thus results may not be fully generalizable to TBI patients in other Central and South American countries. Future investigators are encouraged to collect data from multiple sites in Central and South America.

Another limitation is the lack of investigation of TBI severity on family dynamics, patient SOC, and patient mental and physical HRQL. As injury severity and HRQL have shown diverging results such that Jacobsson, Westerberg, and Lexell [30] found lower HRQL in mild TBI, yet Andelic and colleagues [57] found lower HRQL for individuals with severe TBI, so future investigations are encouraged to investigate any possible differences based on TBI severity. Unfortunately, indices of injury severity commonly used in the United States, such as Glasgow Coma Scale, are not collected regularly in Latin America. As a result, due to a lack of standardization, injury severity and classification could not be accounted for in the current study.

Finally, despite the inferences of causal preponderance that cross-lagged panel analyses allow, in order definitively to prove causation in the relationships found among the variables in this study, experimental methodology is ultimately necessary. Despite the mounting causal evidence shown in this longitudinal design, future research should engage in experimental studies that intervene with family dynamics to determine whether those changes in family dynamics then produce changes in patient SOC and mental and physical HRQL.

## 5. Conclusions

The current study adds to the field’s understanding of the relationships among healthy family dynamics, SOC, and mental and physical HRQL among a sample of TBI patients across three sites and two countries in Latin America over the first four months after injury. It is the first study to investigate the indirect effect of healthy family dynamics on patient mental and physical HRQL via patient SOC, the first to do so in Latin America and over time and also the first to examine the cross-lagged effects of these variables. As familism is an important and crucial value in Latin American culture emphasis on family dynamics in the relation to health outcomes is of vital importance [22]. The rehabilitation workforce is encouraged to assess for family dynamics and patient SOC at the time of TBI hospitalization and throughout recovery, in order to intervene with variables that may eventually improve patient mental and physical HRQL.

## Figures and Tables

**Table 1:** Patient Characteristics.

Variable	Category	(*n* = 109)
Age, *M* (*SD*)		35.87 (14.08)
Years of Education, *M* (*SD*)		9.99 (3.55)
Days in Hospital		20.78 (28.95)
Month Between Injury and Baseline Data Collection, *M* (*SD*)		1.85 (3.06)
Site, *n* (%)	Neiva, Colombia	20 (18.3)
	Cali, Colombia	21 (19.3)
	Mexico City, Mexico	68 (62.4)
TBI Patient Sex, *n* (%)	Male	90 (82.6)
	Female	19 (17.4)
Marriage Status Post-Injury, *n* (%)	Single	48 (44)
	Married	27 (24.8)
	Divorced	2 (1.8)
	Separated	2 (1.8)
	Widowed	2 (1.8)
	Open Relationship	27 (24.8)
	Other	1 (0.9)
TBI Cause, *n* (%)	Automobile Accident	12 (11)
	Motorcycle Accident	41 (37.6)
	Bicycle Accident	3 (2.8)
	Pedestrian accident	7 (6.4)
	Gunshot Wound	2 (1.8)
	Act of Violence	17 (15.6)
	Sports Injury/Accident	1 (0.9)
	Fall	23 (21.1)
	Other	3 (2.8)
Pre-Injury Employment Status, *n* (%)	Full-Time Employment	66 (60.6)
	Part-Time Employment	17 (15.6)
	Homemaker	8 (7.3)
	Unemployed	7 (6.4)
	Student	9 (8.3)
	Pension	1 (0.9)
	Retired	1 (0.9)

**Table 2:** Overall Correlation Matrix.

Study Variables	1	2	3	4	5	6	7	8	9	10	11
1. Physical HRQL Time 1											
2. Physical HRQL Time 2	0.552 **										
3. Physical HRQL Time 3	0.537 **	0.898 **									
4. Mental HRQL Time 1	0.656 **	0.544 **	0.549 **								
5. Mental HRQL Time 2	0.360 **	0.755 **	0.696 **	0.669 **							
6. Mental HRQL Time 3	0.399 **	0.733 **	0.793 **	0.637 **	0.867 **						
7. SOC Time 1	0.213 *	0.368 **	0.448 **	0.524 **	0.491 **	0.519 **					
8. SOC Time 2	0.08	0.453 **	0.412 **	0.273 **	0.557 **	0.490 **	0.591 **				
9. SOC Time 3	0.174	0.410 **	0.481 **	0.365 **	0.504 **	0.596 **	0.694 **	0.740 **			
10. Healthy Family Dynamics Time 1	0.153	0.357 **	0.455 **	0.356 **	0.432 **	0.532 **	0.466 **	0.377 **	0.441 **		
11. Healthy Family Dynamics Time 2	0.151	0.334 **	0.371 **	0.214 *	0.351 **	0.389 **	0.333 **	0.321 **	0.439 **	0.730 **	
12. Healthy Family Dynamics Time 3	0.156	0.317 **	0.367 **	0.169	0.332 **	0.411 **	0.212 *	0.302 **	0.387 **	0.612 **	0.782 **

Note. * *p* < 0.05. ** *p* < 0.01. HRQL = Health-Related Quality of Life; SOC = Sense of Coherence.
